# Supplementary material for: Intestinal Mucosal Barrier Improvement with Prebiotics: Histological Evaluation of Longish Glucomannan Hydrolysates-Induced Innate T Lymphocyte Activities in Mice
Source: Nutrients. 2022 May 26;14(11):2220. doi: 10.3390/nu14112220 (PMC9182621; doi:10.3390/nu14112220)
Supplement: Supplementary file 1 [file nutrients-14-02220-s001.zip › nutrients-1732579-supplementary.pdf]

Supplementary Figure

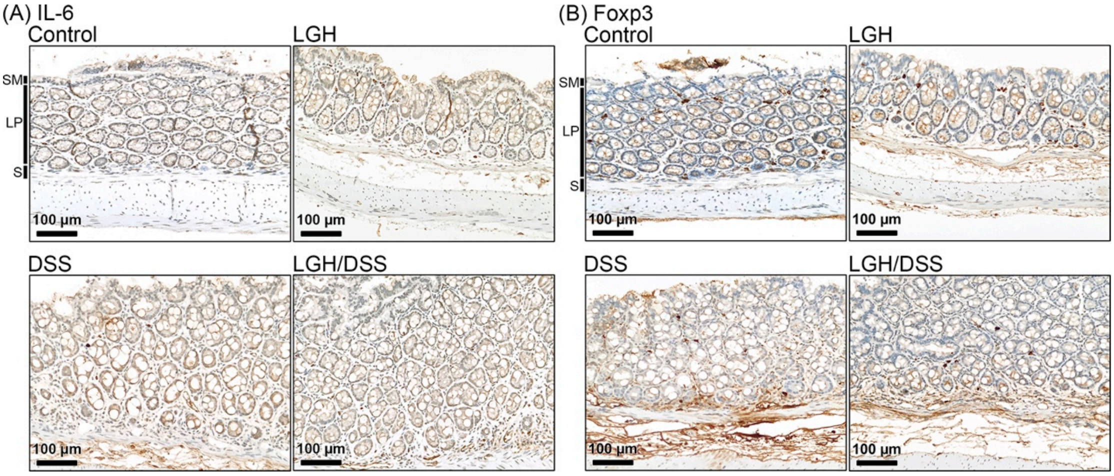

Figure S1. Representative immunohistochemical staining for the expression of IL-6 and Foxp3 in the colons of different groups. Colon tissue sections from control group (upper left panel), LGH group (upper right panel), DSS group (lower left panel), and LGH/DSS group (lower right panel) were stained with IL-6 (A) and Foxp3 (B). SM, superficial mucosa; LP, laminar propria; S, submucosa. DSS, dextran sodium sulfate; LGH, longish glucomannan hydrolysates. Scale bar, 100 μm.

Supplementary Tables

Table S1. IHC scoring scheme for cytokine expression.

Signal intensity		Grade I	Cell expansion		Grade II
**Reactivity**	**Chromophile**		**Extent**	**Range**	
Negative	-	0	Normal	< 10%	0
Light	+	1	Rare	10%–50%	1
Heavy	++	2	Frequent	> 50%	2

Table S2. Histological scoring scheme for colitis.

Feature	Grade	Criterion
Inflammatory severity	0	None
	1	Slight
	2	Moderate
	3	Severe
Inflammatory extent	0	None
	1	Mucosa
	2	Mucosa and submucosa
	3	Transmural
Epithelium regeneration	0	Complete regeneration
	1	Partial regenerative of gland
	2	Minimal regenerative change
	3	No tissue repair
Crypt damage	0	None
	1	Basal 1/3 damaged
	2	Basal 2/3 damaged
	3	Only surface epithelium intact
	**Grade**	**Area**
Involvement	1	0%–25%
	2	26%–50%
	3	51%–75%
	4	76% –100%

Histological criteria were used to evaluate the inflammation. Involvement in quarter of 1–4 described the area percentage of the sample involved.

Table S3. Immunohistochemical evaluation of cytokine expression in colonic mucosa/submucosa.

Cytokine	Location	Control					DSS					LGH					LGH/DSS				
		**0**	**1**	**2**	**3**	**4**	**0**	**1**	**2**	**3**	**4**	**0**	**1**	**2**	**3**	**4**	**0**	**1**	**2**	**3**	4
IL-6	Mucosal surface	9	18	3	0	0	0	6	7	12	5	17	9	4	0	0	21	9	0	0	0
	Lamina propria	0	12	16	2	0	0	8	12	6	4	0	19	11	0	0	0	21	5	4	0
	Submucosa	3	3	16	8	0	0	0	1	19	10	4	10	14	2	0	1	3	13	13	0
TNF-α	Mucosal surface	7	11	12	0	0	0	2	3	10	15	2	10	17	1	0	5	9	14	2	0
	Lamina propria	2	23	5	0	0	0	0	8	11	11	3	16	11	0	0	2	17	10	1	0
	Submucosa	25	4	1	0	0	0	1	17	6	6	26	4	0	0	0	15	10	5	0	0

Total 30 sections with IL-6 or TNF-α immunoactivity were randomly selected from colon samples of each group (Control, DSS, LGH, and LGH/DSS). The IHC scoring scheme with levels of 0–4, as listed in Table S1, was evaluated. DSS, dextran sodium sulfate; LGH, longish glucomannan hydrolysates.
